# Does circadian dysrhythmia drive the switch into high‐ or low‐activation states in bipolar I disorder?

**DOI:** 10.1111/bdi.13304

**Published:** 2023-02-23

**Authors:** Ian B. Hickie, Kathleen R. Merikangas, Joanne S. Carpenter, Frank Iorfino, Elizabeth M. Scott, Jan Scott, Jacob J. Crouse

**Affiliations:** ^1^ Youth Mental Health and Technology Team, Brain and Mind Centre, Faculty of Medicine and Health University of Sydney New South Wales Sydney Australia; ^2^ Genetic Epidemiology Research Branch, Division of Intramural Research Program National Institute of Mental Health Bethesda Maryland USA; ^3^ Institute of Neuroscience Newcastle University Newcastle upon Tyne UK; ^4^ Norwegian University of Science and Technology Trondheim Norway; ^5^ Université de Paris Paris France

**Keywords:** bipolar disorders, circadian rhythms, mood disorders

## Abstract

**Objectives:**

Emerging evidence suggests a role of circadian dysrhythmia in the switch between “activation” states (i.e., objective motor activity and subjective energy) in bipolar I disorder.

**Methods:**

We examined the evidence with respect to four relevant questions: (1) Are natural or environmental exposures that can disrupt circadian rhythms also related to the switch into high‐/low‐activation states? (2) Are circadian dysrhythmias (e.g., altered rest/activity rhythms) associated with the switch into activation states in bipolar disorder? (3) Do interventions that affect the circadian system also affect activation states? (4) Are associations between circadian dysrhythmias and activation states influenced by other “third” factors?

**Results:**

Factors that naturally or experimentally alter circadian rhythms (e.g., light exposure) have been shown to relate to activation states; however future studies need to measure circadian rhythms contemporaneously with these natural/experimental factors. Actigraphic measures of circadian dysrhythmias are associated prospectively with the switch into high‐ or low‐activation states, and more studies are needed to establish the most relevant prognostic actigraphy metrics in bipolar disorder. Interventions that can affect the circadian system (e.g., light therapy, lithium) can also reduce the switch into high‐/low‐activation states. Whether circadian rhythms mediate these clinical effects is an unknown but valuable question. The influence of age, sex, and other confounders on these associations needs to be better characterised.

**Conclusion:**

Based on the reviewed evidence, our view is that circadian dysrhythmia is a plausible driver of transitions into high‐ and low‐activation states and deserves prioritisation in research in bipolar disorders.

## 1 INTRODUCTION

One of the most important processes for discovery and genuine therapeutic advances in clinical medicine is the iterative cycle between observed clinical phenotypes and delineation of putative pathophysiological mechanisms. In clinical psychiatry, only rarely has this iterative process led to genuine breakthroughs. Limited examples do exist for various kinds of mood disorders, including: (1) delineation of “vascular depression” among older adults with a first onset of depression in later life after specific cerebrovascular lesions^1^; (2) onset of depressive disorders after specific infections^2^ or the introduction of immune‐active therapies^3^; and (3) recognition of “atypical” mood disorders in young adults with antineuronal antibodies.^4^ The latter discovery has led to the use of immune therapies targeted to underlying pathophysiology.^5^ One pathophysiological mechanism that may be relevant to major mood disorders, and particularly bipolar I disorder (BD‐I), is disturbance in the 24‐h circadian system.^6^, ^7^, ^8^

In recent years, the field of “Circadian Medicine” has made major contributions to our scientific understanding and treatment of many illnesses, including dementia, cancer, and cardiovascular disease,^9^ with the *Nobel Prize in Physiology or Medicine* awarded for the delineation of the molecular mechanisms controlling the 24‐h circadian clock. While the relevance of perturbed circadian rhythms (or “dysrhythmias”) to mood disorders has been long recognised,^10^ more recent neurobiological, interventional, and longitudinal studies justify a more considered re‐evaluation of the potentially *causative* nature of these associations, specifically regarding whether circadian dysrhythmias *drive* the switch into “activation” states in BD‐I. By “activation” states, we refer to the linked phenomena of objective motor activity and subjective energy, and critically, to intraindividual change in these phenomena.^11^

Conceptually, we first deconstruct the category of BD‐I into two broad constituent states and a transition process: (1) a “high motor activation” (manic) phase; (2) a “low motor activation” (depressed) phase; and (3) a tendency to switch between these abnormal “activation” states. In this framework, the primary focus is on activation, while subjective mood is considered separately.^11^ This phenomenological deconstruction is supported by factor analytic,^11^ family,^12^, ^13^, ^14^ and clinical studies, which indicate that activation and mood may vary independently. Such decoupling of activation and mood is exemplified in mixed states (e.g., high activation and low mood in “dysphoric mania”; high activation and low mood in “agitated depression”); although these are not the focus here.^15^ Our focus on activation as a core feature of BD‐I is consistent with DSM‐5's recognition of changes in activity and energy (alongside mood) as a criterion A symptom, and recent publications about the potential primacy of activation in BD, which have highlighted the need for investigation of the physiological substrates of the transition between activation states, to which circadian rhythms may be relevant.^11^, ^16^, ^17^, ^18^, ^19^, ^20^

“Circadian dysrhythmias” (variously defined) are commonly reported in individuals with BD‐I and are associated with a range of core clinical features.^21^ We use *circadian dysrhythmia* as an umbrella construct that represents a “change in one or more aspects of a circadian cycle's morphology”.^22^ We recognise that multiple phenotypes could be relevant to activation states in BD (e.g., phase delay, phase advance, high fragmentation, blunted amplitude, internal desynchrony, arrhythmia), and we note that most studies in this area have examined actigraphy‐based estimates of phase, amplitude, and fragmentation. In this article, we consider whether circadian dysrhythmias may be a causative *driver* of the switch into high and low motor activation states in BD‐I, rather than mere correlates or epiphenomena.

We undertook a selective/narrative review of the evidence of the association between circadian dysrhythmias and changes in activation states in BD‐I. We summarise the evidence in tables that examine four key questions, and finally discuss the available evidence together. Our target questions were as follows:

1. Are natural or experimental exposures that are associated with circadian dysrhythmia also associated with the switch into high‐ or low‐activation states? (Table 1)
2. Are circadian dysrhythmias associated with the switch into high‐ or low‐activation states? (Table 2)
3. Do interventions that affect the circadian system also affect high‐ or low‐activation states? (Table 3)
4. Are associations between circadian dysrhythmias and high‐ or low‐activation states confounded by “third” factors? (Table 4)

**TABLE 1:** Associations between transitions into high‐ or low‐activation states and natural or experimental exposures capable of disrupting the circadian system.

Switch into high activation (“Mania”)	Switch into low activation (“Depression”)	Comments and potential confounds
**Experimental conditions** *Time zone travel* Some evidence of increased risk of mania/hypomania after eastward travel^59^ 2 *Experimental misalignment and/or sleep loss* In patients with BD‐I or BD‐II, one night of sleep deprivation was associated with a switch from depression into mania (*n* = 4) or hypo/mania (*n* = 3)^60^	**Experimental conditions** *Time zone travel* Some evidence of increased risk of depression after westward travel^59^ 2 *Experimental misalignment and/or sleep loss* Among healthy controls, simulated shift work led to depressive symptoms^61^ Among healthy controls, experimental sleep deprivation led to depressed mood^62^, ^63^, ^64^	Few published studies of time zone travel; re retrospective, correlational designs Small sample size makes conclusions regarding sleep deprivation in BD difficult^60^ Lack of objective measurement of circadian dysrhythmias
**Environmental changes** *1. Seasonality* Spring–Summer associated with increased risk of switch to mania/hypomania^65^, ^66^ *2. Light exposure* Daytime light exposure metrics not associated with switch into manic/hypomanic/mixed episodes^67^ Variations in seasonal solar insolation associated with earlier age of onset of BD‐I, with a smaller effect for initial episode of mania compared to initial episode of depression^35^	**Environmental changes** *1. Seasonality* Early winter associated with increased risk of switch to depression^65^ *2. Light exposure* Daytime light exposure metrics (e.g.longer time above 1000 lux, higher average illuminance) associated with decreased risk of transition into depression^67^ Variations in seasonal solar insolation associated with earlier age of onset of BD‐I, with a larger effect for initial episode of depression compared to initial episode of mania^35^	Some studies do not observe a seasonal pattern in BD Other meteorological factors (e.g., ambient temperature) may partly explain the link between seasonality and activation states Lack of objective measurement of circadian changes alongside changes in seasons, photoperiod, and or light exposure
**Substance use** Stimulants associated with precipitation of mania/hypomania^68^ Evidence of cannabis use preceding mania/hypomania^69^, ^70^ Illicit drugs (broadly defined) reported as a trigger of mania/hypomania^71^ Corticosteroid medications (e.g., prednisone) are reported in case studies as a trigger of mania/hypomania^38^, ^72^	**Substance use** Evidence of alcohol use preceding depression^69^; however, not in all studies^70^	Links between substance use and activation states may be due to non‐circadian effects (e.g., effects on dopamine/glutamate) Lack of objective measurement of circadian dysrhythmias
**Childbirth** Puerperal psychosis follows 20%–30% of births among women with a history of BD^73^ Women reporting sleep loss triggering episodes of mania more likely to have experienced an episode of puerperal psychosis^74^	**Childbirth** Increased risk of depression during post‐partum period among women with BD‐I/BD‐II^75^	Links between childbirth and activation states may be due to non‐circadian effects (e.g., endocrine changes, sleep loss without circadian changes) Several studies hampered by cross‐sectional designs^74^ Lack of objective measurement of circadian dysrhythmias
**Infection** Individuals hospitalised with acute mania have increased history of prescriptions for antimicrobial infections^76^	**Infection** Higher risk of depression after infection (e.g., Epstein–Barr, Coxiella burnetiid, Ross River virus, West Nile virus, Hepatitis C)^2^, ^77^, ^78^, ^79^ Also mixed findings (e.g., influenza, coronaviruses)^80^	Links between infection and activation states may be due to non‐circadian effects (e.g., immune activation)^76^ Lack of objective measurement of circadian dysrhythmias

**TABLE 2:** Associations between estimated circadian dysrhythmias and the course of low‐ and high‐activation states.

Switch into high activation (“Mania”)	Switch into low activation (“Depression”)	Comments and potential confounds
**Rest‐activity rhythms** Intra‐daily variability of rest/activity rhythm associated with relapse (manic, hypomanic, or mixed episode)^52^ Null finding of a within‐person association between rest/activity rhythm and days spent in a manic/hypomanic episode^53^	**Rest‐activity rhythms** Rest/activity rhythms associated with relapse of depressive episode^52^ Within‐person association between rest/activity rhythm and days spent in a depressive episode^53^	Actigraphy is not a direct measure of internal circadian timing and is subject to masking effects (e.g., social activity, work schedules, volitional behaviour) Few studies have examined prospective associations of actigraphy‐estimated circadian dysrhythmias and switches into mania and/or depression
**Physiological rhythms** Melatonin rhythm did not differ between patients in mania, depression, or euthymia^81^ No change in melatonin or cortisol rhythm across manic, depressed, or euthymic phases (in a single patient)^82^	**Physiological rhythms** Melatonin rhythm did not differ between patients in mania, depression, or euthymia^81^ No change in melatonin or cortisol rhythm across manic, depressed, or euthymic phases (in a single patient)^82^	Conclusions are hampered by small sample size (patients; *n* = 9)^81^ and single patient case studies^82^
**Social rhythms** Social rhythm irregularity associated with transition to mania/hypomania among a bipolar spectrum sample^83^ Social rhythm disturbance associated with transition into a manic episode^84^, ^85^	**Social rhythms** Social rhythm irregularity associated with transition to depression among a bipolar spectrum sample^83^ Lack of association between social rhythm disturbance and transition into a depressive episode^84^, ^85^	Social rhythms are not a direct measure of internal circadian timing Conclusions hampered by small sample sizes in some studies^84^ No study has examined whether circadian dysrhythmia mediates the association between social rhythm disruption and relapse/switch

**TABLE 3:** Associations between interventions that act on the circadian system and high‐ and low‐activation states in BD‐I.

Switch into high activation (“Mania”)	Switch into low activation (“Depression”)	Comments and potential confounds
**Lithium** Highly effective for reducing acute mania^86^, ^87^, ^88^ Highly effective for reducing relapse of mania^88^, ^89^, ^90^	**Lithium** Some benefit for reducing acute depression^88^ Some benefit for reducing relapse of depression^88^	Lithium's *primary* prophylactic effects may be non‐circadian^91^ Only preliminary evidence that lithium has circadian effects in humans^92^ No human study has examined whether changes in circadian dysrhythmia mediate lithium's effects
**Melatonergic agents** Unclear effects for reducing acute mania Agents such as ramelteon may not be effective for reducing relapse of mania^47^	**Melatonergic agents** Agomelatine has some benefit for reducing acute depression,^41^, ^42^ but also negative findings^46^ Agomelatine has some benefit for reducing relapse of depression^43^ Ramelteon may not be effective for reducing relapse of depression^47^	Only preliminary evidence that agomelatine has circadian effects^49^ Agomelatine was adjunctive, with patients co‐medicated (e.g., lithium, valpromide)^41^, ^46^ Several studies limited by small sample^42^ or open label designs^41^ Some studies used a combined relapse measure for mania or depression^43^, ^47^
**Bright light therapy** Unclear effects for reducing acute mania or relapse of mania	**Bright light therapy** Some benefit for reducing acute depression in some studies,^44^, ^45^ but not others^48^ May not be effective for reducing relapse of depression^44^, ^45^	Effects of bright light therapy on acute depression could be non‐circadian (e.g., direct effects on mood circuits)^29^
**Dark therapy** Associated with reducing acute mania in some studies,^93^, ^94^, ^95^ but not others^96^ Unclear effects for relapse of mania	**Dark therapy** Does not appear to be associated with reduction in depression^26^, ^96^	Conclusions hampered by small sample sizes^93^
**Sleep deprivation (or wake therapy)** Not recommended for acute mania^26^ Unclear effects for relapse of mania	**Sleep deprivation (or wake therapy)** Some benefit for reducing acute depression^26^	Effects of sleep deprivation may be non‐circadian (e.g., synaptic plasticity)

**TABLE 4:** Are associations between circadian dysrhythmia and high‐ and low‐activation states explained by a third common factor?

High activation (“Mania”)	Low activation (“Depression”)	Comments and potential confounds
**Age and sex** Several studies show associations between BD‐I and circadian dysrhythmia independent of age and sex^18^ One study shows an association between transition into mania and objective circadian dysrhythmia, adjusted for age/sex^52^	**Age and sex** Several studies show associations between BD‐I and circadian dysrhythmia independent of age and sex^18^ One study shows an association between transition into depression and objective circadian dysrhythmia, adjusted for age/sex^52^	More studies needed that account for age and sex
**Neurodevelopmental impairment** Unclear (limited evidence)	**Neurodevelopmental impairment** Circadian rhythm sleep/wake disturbance (e.g., delayed sleep phase) are observed in samples excluding participants with low IQ or history of head injury^97^	This evidence comes from a study of a mixed diagnostic sample^97^ Few studies appear to examine these types of impairments
**Genetic factors** Mixed evidence regarding associations of circadian genes and BD (broadly defined)^33^, ^98^ Some evidence for genetic associations among BD‐I pedigrees and circadian dysrhythmia (blunted rest/activity rhythm)^99^ No association between polygenic risk score (PRS) for low amplitude of rest/activity rhythm and BD, but a significant association with mood instability^58^	**Genetic factors** Mixed evidence regarding associations of circadian genes and BD (broadly defined)^33^, ^98^ Some evidence for an association among a seasonal pattern of depressive episodes and circadian genes^100^	Lack of evidence regarding associations of manic and depressed states and circadian dysrhythmias, accounting for genetic factors

Our selection of evidence for this review was based in part on our clinical experience and associated priors (e.g., potential links between infection, circadian disturbance, and BD), literature searches using key terms (e.g., “circadian rhythms”, “actigraphy”, “motor activity”, “bipolar”, “mania”, “depression”, “clinical trial”), and searches through our personal files. We aimed to primarily include studies that explicitly report on motor activation; however, we also include studies that examined biological circadian rhythms (e.g., melatonin), and studies of patients that may not have been included based on motor activation. We note that the scope of this review is focused, and we point interested readers to a recent comprehensive review^8^ of additional areas that fell outside our scope (e.g., preclinical cellular and animal models, molecular genetic studies, studies not focused on motor activation).

## 2 DISCUSSION

In this narrative review, we have outlined evidence relevant to our hypothesis that circadian dysrhythmias have the capacity to drive switches into high‐ or low‐activation states in BD‐I. While we postulate that circadian dysrhythmias *can* causally increase the likelihood of these transitions, it is highly likely that this risk is conditional on other risk factors, such as genetic risk for BD or biological and environmental factors (e.g., sensitivity to light, local variation in light or other seasonally patterned factors). In other words, circadian dysrhythmias may be a *component cause* of variation in the course of activation states in BD‐I.^23^ It is also important to note that while we focused our efforts on bipolar disorders (and BD‐I specifically), there is evidence that circadian dysrhythmias may play a role in the course of other mental disorders (e.g., psychotic disorders, depressive disorders),^24^, ^25^ and there are important limitations which should be addressed in future research. Most studies reviewed used correlational designs and lacked circadian recordings alongside the proposed triggers (Table 1) and in treatment studies (Table 3). Very few studies used direct measures of circadian timing, and conclusions about central timing based on real‐world actigraphy data are hampered by their limited concordance with central measures (e.g., dim‐light melatonin onset) and controlled settings (e.g., constant routine), and the susceptibility of actigraphy to various masking effects (e.g., voluntary behaviour, social activity, work schedules). Finally, we acknowledge that our review covers a focused area (human studies relevant to motor activation in BD‐I) in the wider context of chronobiology and mental disorders, for which there are reviews^8^, ^26^ on preclinical models,^27^, ^28^, ^29^ neurobiology,^7^, ^30^ phenomenology,^31^ measurement,^17^, ^31^ pharmacology,^28^ modelling,^32^ and genetics.^33^, ^34^

In the context of these limitations, we conclude there is: (1) reasonable support for the notion that natural or experimental events, circumstances, or factors that can perturb circadian rhythms can also precipitate switches into activation states; (2) reasonable support for the notion that interventions that can act directly on the circadian system can also affect activation states (with much more work needed to test whether circadian changes mediate these clinical effects); (3) limited (but emerging) support for the notion that objective or estimated circadian dysrhythmias are associated with transitions into activation states; and (4) limited evidence for the notion that associations between circadian dysrhythmias and activation states are not confounded by third factors including, but not limited to, age or development stage, sex, genetics, or neurodevelopmental injury or impairment. We will now discuss these findings with respect to their potential meaning, other limitations, and areas for further study.

To begin with, a range of studies of quite different natural or experimental factors that have all been shown to be associated with circadian dysrhythmia demonstrate a pattern of precipitation of the switch to high‐ and low‐activation states, with some factors possibly associated also with first onset of these states (e.g., high variation in solar insolation.^35^) Some of these factors—like seasonal changes in solar insolation, experimental sleep deprivation, and childbirth—appear to have quite reliable effects, while others, like substance use and infection, that have low absolute risk of transition to high‐ or low‐activation states, have nonetheless been shown to be related to circadian dysrhythmia.^36^ Of these, those that have fundamental biological links to the circadian system and also clinical or epidemiological links to activation states, such as variation in photoperiod or solar insolation across the seasons (and potentially *rate of change* in these light‐related measures^37^), are the most convincing. A key limitation of our grouping these different exposures together is that each of these factors are highly likely to have *non‐circadian* effects that may be more directly linked to depressive and manic states—for example, amphetamine‐type stimulants affecting dopaminergic function, direct effects of light exposure on brain mood circuits, major endocrine changes accompanying childbirth, among others.^29^, ^38^ By linking them together in a conceptual framework of circadian dysrhythmia, we hope to generate interest in these testable hypotheses in studies of circadian rhythms and activation states in BD. Another limitation with respect to our research question, and the main barrier to evaluating a causative effect of circadian dysrhythmia on transitions into activation states following these events, is the lack of objective circadian measures contemporaneous with these exposures. To provide a clearer answer to this question, future studies could use ambulatory measures (e.g., actigraphy) or controlled laboratory meaures (e.g., dim‐light melatonin onset) to estimate changes in circadian rhythms during the transition through certain exposures (e.g., comparing patients living in regions with high versus low seasonal variation in solar insolation) to test whether circadian dysrhythmias mediate transitions into mania/depression. Altogether, the natural and experimental exposures reviewed provide some suggestion that the onset of circadian dysrhythmia may precede the transition into high (manic) and low (depression) activation states and should provoke study of these exposures using prospective, hypothesis‐testing designs.

The next most convincing factor is that interventions that target the circadian system (e.g., bright light therapy, dark therapy, melatonergic agents, lithium) can lead to resolution of mania or depression. While lithium has strong evidence for amelioration of mania, and reduction in relapse, its exact effects on circadian systems remain to be fully characterised. However, there is substantial evidence of the circadian effects of lithium across a range of human and animal studies (e.g., cultured cells, healthy humans, animal models, patient‐derived fibroblasts).^27^, ^39^, ^40^ For depression, there is also evidence that melatonergic agents (e.g., agomelatine) and bright light therapy may have significant benefits^41^, ^42^, ^43^, ^44^, ^45^; however, this evidence is quite mixed,^44^, ^45^, ^46^, ^47^, ^48^ suggesting further investigation of treatment response (e.g., response/non‐response subgroups) may be needed. Progress here now depends on better clinical trial designs that stratify mood disorders cohorts according to their circadian characteristics or include circadian‐based measures (e.g., actigraphy, melatonin rhythms) within repeated assessment schedules. Such approaches would then permit direct testing of the potential mediating effects of circadian rhythms on changes in activation states. Some studies of agomelatine^49^ and ketamine^50^ have adopted these approaches, as have other ongoing clinical trials (e.g., brexpiprazole).^51^

The sparse evidence‐base for examining our two remaining questions (Tables 2 and 4) means that only limited conclusions are possible. With regards to the postulate that ongoing circadian dysfunction is associated with ongoing illness, only a small number of studies have examined prospective associations between circadian markers and mania/depression. One study reported that circadian rest/activity markers were associated with relapse of manic and/or depressive episodes,^52^ and another reported an association between rest/activity rhythms and days in a depressive episode.^53^ While one study reported a longitudinal association between change in dim‐light onset melatonin rhythms and depressive symptoms,^49^ the overall literature regarding relationships between biological rhythms and manic and depressive states is mixed. Finally, the influence of possible “third factors” on the observed associations between circadian perturbations and mania and depression remains an unexplored area. Few studies have examined the impacts of factors such as age, neurodevelopmental stage, and biological sex on these associations. Several recent studies have reported overlap between genetic variants associated with BD and sleep and circadian phenotypes.^54^, ^55^, ^56^, ^57^ For example, a study using a polygenic risk score (PRS) for actigraphy‐based relative amplitude found no association with BD, but a significant association with mood instability.^58^ A genome‐wide association study (GWAS) of >40,000 cases with BD reported positive genetic correlations between BD and sleep phenotypes (insomnia, daytime sleepiness, sleep duration, daytime napping, “getting up in morning”) but not chronotype.^56^ Moreover, there were bidirectional (putatively causal) associations between BD and longer sleep duration, and a unidirectional (putatively causal) association between BD and a lower likelihood of being a morning person.^56^ However, some GWASs have reported mixed findings, with significant genetic correlations between BD and some sleep or circadian phenotypes (e.g., sleep duration) but not others (e.g., insomnia, chronotype).^55^ A case–control study observed differential associations among BD subtypes and polygenic liability to sleep duration and insomnia, but not for chronotype.^57^ While mounting evidence shows shared genetic risk for BD and *sleep* phenotypes, we note that the role of genetic and biological factors (e.g., neurodevelopment) linking *circadian* phenotypes and BD is less clear, and should be explored further in genetic, twin, family, and prospective studies, particularly those following individuals through developmental phases of peak risk of BD.

Considering the evidence reviewed, our view is that circadian dysrhythmia is a plausible driver of transition into both high‐ and low‐activation states in BD‐I. While we have mostly focused here on the associations between circadian dysrhythmias and illness course in established BD‐I, there is great interest in whether these disturbances may also play a role in the aetiology of mania and depression, an under‐researched question. Our hypothesis that circadian dysrhythmias can drive the transition into high‐ and low‐activation states in BD‐I, if true, has implications for indicated prevention, early intervention, and personalised treatment choices, and we believe it requires prioritisation in clinical research. We believe that the best tests of causative effects of circadian dysrhythmia on activation states in BD‐I will come from genetically‐informative twin and family studies, observational studies, and causal‐interventionist clinical trials.

## CONFLICT OF INTEREST STATEMENT

IBH is the Co‐Director, Health and Policy at the Brain and Mind Centre (BMC) University of Sydney, Australia. The BMC operates an early‐intervention youth services at Camperdown under contract to headspace. Professor Hickie has previously led community‐based and pharmaceutical industry‐supported (Wyeth, Eli Lily, Servier, Pfizer, AstraZeneca) projects focused on the identification and better management of anxiety and depression. He is the Chief Scientific Advisor to, and a 3.2% equity shareholder in, InnoWell Pty Ltd. InnoWell was formed by the University of Sydney (45% equity) and PwC (Australia; 45% equity) to deliver the $30 M Australian Government‐funded Project Synergy (2017–20) and to lead transformation of mental health services internationally through the use of innovative technologies. EMS is the Medical Director, Young Adult Mental Health Unit, St Vincent's Hospital Darlinghurst, Discipline Leader of Adult Mental Health, School of Medicine, University of Notre Dame, Research Affiliate, The University of Sydney and Consultant Psychiatrist. She has received honoraria for educational seminars related to the clinical management of depressive disorders supported by Servier and Eli‐Lilly pharmaceuticals. She has participated in a national advisory board for the antidepressant compound Pristiq, manufactured by Pfizer. She was the National Coordinator of an antidepressant trial sponsored by Servier. The other authors declare no competing interests.

## Data Availability

Data sharing is not applicable to this article as no new data were created or analyzed in this study.

## References

[1] Naismith S , Norrie L , Mowszowski L , Hickie I . The neurobiology of depression in later‐life: clinical, neuropsychological, neuroimaging and pathophysiological features. Prog Neurobiol. 2012;98:99‐143. 22609700 10.1016/j.pneurobio.2012.05.009

[2] Hickie I , Davenport T , Wakefield D , et al. Post‐infective and chronic fatigue syndromes precipitated by viral and non‐viral pathogens: prospective cohort study. BMJ. 2006;333(7568):575. 16950834 10.1136/bmj.38933.585764.AE PMC1569956

[3] Capuron L , Miller AH . Cytokines and psychopathology: lessons from interferon‐α. Biol Psychiatry. 2004;56(11):819‐824. 15576057 10.1016/j.biopsych.2004.02.009

[4] Schou MB , Sæther SG , Drange OK , et al. A prospective three‐year follow‐up study on the clinical significance of anti‐neuronal antibodies in acute psychiatric disorders. Sci Rep. 2019;10(1):35. 31896766 10.1038/s41598-019-56934-6 PMC6940359

[5] Pape K , Tamouza R , Leboyer M , Zipp F . Immunoneuropsychiatry ‐ novel perspectives on brain disorders. Nat Rev Neurol. 2019;15(6):317‐328. 30988501 10.1038/s41582-019-0174-4

[6] Crouse JJ , Carpenter JS , Song YJC , et al. Circadian rhythm sleep‐wake disturbances and depression in young people: implications for prevention and early intervention. Lancet Psychiatry. 2021;8(9):813‐823. 34419186 10.1016/S2215-0366(21)00034-1

[7] Logan RW , McClung CA . Rhythms of life: circadian disruption and brain disorders across the lifespan. Nat Rev Neurosci. 2019;20(1):49‐65. 30459365 10.1038/s41583-018-0088-y PMC6338075

[8] McCarthy MJ , Gottlieb JF , Gonzalez R , et al. Neurobiological and behavioral mechanisms of circadian rhythm disruption in bipolar disorder: a critical multi‐disciplinary literature review and agenda for future research from the ISBD task force on chronobiology. Bipolar Disord. 2022;24(3):232‐263. 34850507 10.1111/bdi.13165 PMC9149148

[9] Allada R , Bass J . Circadian mechanisms in medicine. N Engl J Med. 2021;384(6):550‐561. 33567194 10.1056/NEJMra1802337 PMC8108270

[10] Richter CP . Biological clocks in medicine and psychiatry: shock‐phase hypothesis. Proc Natl Acad Sci U S A. 1960;46(11):1506‐1530. 16590778 10.1073/pnas.46.11.1506 PMC223074

[11] Scott J , Murray G , Henry C , et al. Activation in bipolar disorders: a systematic review. JAMA Psychiat. 2017;74(2):189‐196. 10.1001/jamapsychiatry.2016.3459 28002572

[12] Hickie IB . Evidence for separate inheritance of mania and depression challenges current concepts of bipolar mood disorder. Mol Psychiatry. 2014;19(2):153‐155. 24365866 10.1038/mp.2013.173

[13] Merikangas KR , Cui L , Heaton L , et al. Independence of familial transmission of mania and depression: results of the NIMH family study of affective spectrum disorders. Mol Psychiatry. 2014;19(2):214‐219. 24126930 10.1038/mp.2013.116

[14] Vandeleur CL , Merikangas KR , Strippoli MP , Castelao E , Preisig M . Specificity of psychosis, mania and major depression in a contemporary family study. Mol Psychiatry. 2014;19(2):209‐213. 24126925 10.1038/mp.2013.132

[15] Johnson SL , Morriss R , Scott J , et al. Depressive and manic symptoms are not opposite poles in bipolar disorder. Acta Psychiatr Scand. 2011;123(3):206‐210. 20825373 10.1111/j.1600-0447.2010.01602.x PMC3402361

[16] Merikangas KR , Swendsen J , Hickie IB , et al. Real‐time mobile monitoring of the dynamic associations among motor activity, energy, mood, and sleep in adults with bipolar disorder. JAMA Psychiat. 2019;76(2):190‐198. 10.1001/jamapsychiatry.2018.3546 PMC6439734 30540352

[17] Dunster GP , Swendsen J , Merikangas KR . Real‐time mobile monitoring of bipolar disorder: a review of evidence and future directions. Neuropsychopharmacology. 2020;46:197‐208. 32919408 10.1038/s41386-020-00830-5 PMC7688933

[18] Shou H , Cui L , Hickie I , et al. Dysregulation of objectively assessed 24‐hour motor activity patterns as a potential marker for bipolar I disorder: results of a community‐based family study. Transl Psychiatry. 2017;7(8):e1211. 28892068 10.1038/tp.2017.136 PMC5611716

[19] Scott J , Colom F , Young A , Bellivier F , Etain B . An evidence map of actigraphy studies exploring longitudinal associations between rest‐activity rhythms and course and outcome of bipolar disorders. Int J Bipolar Disord. 2020;8(1):37. 33258017 10.1186/s40345-020-00200-6 PMC7704984

[20] Cassano GB , Rucci P , Benvenuti A , et al. The role of psychomotor activation in discriminating unipolar from bipolar disorders: a classification‐tree analysis. J Clin Psychiatry. 2012;73(1):22‐28. 22316575 10.4088/JCP.11m06946

[21] Melo MCA , Abreu RLC , Linhares Neto VB , de Bruin PFC , de Bruin VMS . Chronotype and circadian rhythm in bipolar disorder: a systematic review. Sleep Med Rev. 2017;34:46‐58. 27524206 10.1016/j.smrv.2016.06.007

[22] McKenna HT , Reiss IK , Martin DS . The significance of circadian rhythms and dysrhythmias in critical illness. J Intensive Care Soc. 2017;18(2):121‐129. 28979558 10.1177/1751143717692603 PMC5606425

[23] Marangoni C , Hernandez M , Faedda GL . The role of environmental exposures as risk factors for bipolar disorder: a systematic review of longitudinal studies. J Affect Disord. 2016;193:165‐174. 26773919 10.1016/j.jad.2015.12.055

[24] Lunsford‐Avery JR , Gonçalves BSB , Brietzke E , et al. Adolescents at clinical‐high risk for psychosis: circadian rhythm disturbances predict worsened prognosis at 1‐year follow‐up. Schizophr Res. 2017;189:37‐42. 28169087 10.1016/j.schres.2017.01.051 PMC5544586

[25] Carpenter JS , Crouse JJ , Scott EM , et al. Circadian depression: a mood disorder phenotype. Neurosci Biobehav Rev. 2021;126:79‐101. 33689801 10.1016/j.neubiorev.2021.02.045

[26] Gottlieb JF , Benedetti F , Geoffroy PA , et al. The chronotherapeutic treatment of bipolar disorders: a systematic review and practice recommendations from the ISBD task force on chronotherapy and chronobiology. Bipolar Disord. 2019;21(8):741‐773. 31609530 10.1111/bdi.12847

[27] McCarthy MJ , Wei H , Marnoy Z , et al. Genetic and clinical factors predict lithium's effects on PER2 gene expression rhythms in cells from bipolar disorder patients. Transl Psychiatry. 2013;3(10):e318. 24150227 10.1038/tp.2013.90 PMC3818008

[28] McCarthy MJ , Wei H , Nievergelt CM , et al. Chronotype and cellular circadian rhythms predict the clinical response to lithium maintenance treatment in patients with bipolar disorder. Neuropsychopharmacology. 2019;44(3):620‐628. 30487653 10.1038/s41386-018-0273-8 PMC6333516

[29] LeGates TA , Fernandez DC , Hattar S . Light as a central modulator of circadian rhythms, sleep and affect. Nat Rev Neurosci. 2014;15(7):443‐454. 24917305 10.1038/nrn3743 PMC4254760

[30] Ketchesin KD , Zong W , Hildebrand MA , et al. Diurnal rhythms across the human dorsal and ventral striatum. Proc Natl Acad Sci U S A. 2021;118(2):e2016150118. 33372142 10.1073/pnas.2016150118 PMC7812799

[31] Murray G , Gottlieb J , Hidalgo MP , et al. Measuring circadian function in bipolar disorders: empirical and conceptual review of physiological, actigraphic, and self‐report approaches. Bipolar Disord. 2020;22:693‐710. 32564457 10.1111/bdi.12963

[32] Bayani A , Hadaeghi F , Jafari S , Murray G . Critical slowing down as an early warning of transitions in episodes of bipolar disorder: a simulation study based on a computational model of circadian activity rhythms. Chronobiol Int. 2017;34(2):235‐245. 28060532 10.1080/07420528.2016.1272608

[33] McCarthy MJ , Nievergelt CM , Kelsoe JR , Welsh DK . A survey of genomic studies supports association of circadian clock genes with bipolar disorder spectrum illnesses and lithium response. PLoS One. 2012;7(2):e32091. 22384149 10.1371/journal.pone.0032091 PMC3285204

[34] McCarthy MJ . Missing a beat: assessment of circadian rhythm abnormalities in bipolar disorder in the genomic era. Psychiatr Genet. 2019;29(2):29‐36. 30516584 10.1097/YPG.0000000000000215

[35] Bauer M , Glenn T , Alda M , et al. Solar insolation in springtime influences age of onset of bipolar I disorder. Acta Psychiatr Scand. 2017;136(6):571‐582. 28722128 10.1111/acps.12772

[36] Hasler BP , Smith LJ , Cousins JC , Bootzin RR . Circadian rhythms, sleep, and substance abuse. Sleep Med Rev. 2012;16(1):67‐81. 21620743 10.1016/j.smrv.2011.03.004 PMC3177010

[37] Rosenthal SJ , Josephs T , Kovtun O , McCarty R . Rate of change in solar insolation is a hidden variable that influences seasonal alterations in bipolar disorder. Brain Behav. 2021;11(7):e02198. 34061463 10.1002/brb3.2198 PMC8323043

[38] Proudfoot J , Doran J , Manicavasagar V , Parker G . The precipitants of manic/hypomanic episodes in the context of bipolar disorder: a review. J Affect Disord. 2011;133(3):381‐387. 21106249 10.1016/j.jad.2010.10.051

[39] Bellivier F , Geoffroy PA , Etain B , Scott J . Sleep‐ and circadian rhythm‐associated pathways as therapeutic targets in bipolar disorder. Expert Opin Ther Targets. 2015;19(6):747‐763. 25726988 10.1517/14728222.2015.1018822

[40] Sanghani HR , Jagannath A , Humberstone T , et al. Patient fibroblast circadian rhythms predict lithium sensitivity in bipolar disorder. Mol Psychiatry. 2021;26(9):5252‐5265. 32404948 10.1038/s41380-020-0769-6 PMC8589670

[41] Calabrese JR , Guelfi JD , Perdrizet‐Chevallier C , Agomelatine Bipolar Study Group . Agomelatine adjunctive therapy for acute bipolar depression: preliminary open data. Bipolar Disord. 2007;9(6):628‐635. 17845278 10.1111/j.1399-5618.2007.00507.x

[42] McElroy SL , Winstanley EL , Martens B , et al. A randomized, placebo‐controlled study of adjunctive ramelteon in ambulatory bipolar I disorder with manic symptoms and sleep disturbance. Int Clin Psychopharmacol. 2011;26(1):48‐53. 20861739 10.1097/YIC.0b013e3283400d35

[43] Norris ER , Karen B , Correll JR , et al. A double‐blind, randomized, placebo‐controlled trial of adjunctive ramelteon for the treatment of insomnia and mood stability in patients with euthymic bipolar disorder. J Affect Disord. 2013;144(1–2):141‐147. 22963894 10.1016/j.jad.2012.06.023

[44] Hirakawa H , Terao T , Muronaga M , Ishii N . Adjunctive bright light therapy for treating bipolar depression: a systematic review and meta‐analysis of randomized controlled trials. Brain Behav. 2020;10(12):e01876. 33034127 10.1002/brb3.1876 PMC7749573

[45] Lam RW , Teng MY , Jung YE , et al. Light therapy for patients with bipolar depression: systematic review and meta‐analysis of randomized controlled trials. Can J Psychiatr. 2020;65(5):290‐300. 10.1177/0706743719892471 PMC7265610 31826657

[46] Yatham LN , Vieta E , Goodwin GM , et al. Agomelatine or placebo as adjunctive therapy to a mood stabiliser in bipolar I depression: randomised double‐blind placebo‐controlled trial. Br J Psychiatry. 2016;208(1):78‐86. 25999335 10.1192/bjp.bp.114.147587

[47] Mahableshwarkar AR , Calabrese JR , Macek TA , et al. Efficacy and safety of sublingual ramelteon as an adjunctive therapy in the maintenance treatment of bipolar I disorder in adults: a phase 3, randomized controlled trial. J Affect Disord. 2017;221:275‐282. 28662460 10.1016/j.jad.2017.06.044

[48] Takeshima M , Utsumi T , Aoki Y , et al. Efficacy and safety of bright light therapy for manic and depressive symptoms in patients with bipolar disorder: a systematic review and meta‐analysis. Psychiatry Clin Neurosci. 2020;74(4):247‐256. 31917880 10.1111/pcn.12976 PMC7187384

[49] Robillard R , Carpenter JS , Feilds K , et al. Parallel changes in mood and melatonin rhythm following an adjunctive multimodal Chronobiological intervention with Agomelatine in people with depression: a proof of concept open label study. Front Psych. 2018;9:624. 10.3389/fpsyt.2018.00624 PMC6297866 30618853

[50] Duncan WCJ , Slonena E , Hejazi NS , et al. Motor‐activity markers of circadian timekeeping are related to Ketamine's rapid antidepressant properties. Biol Psychiatry. 2017;82(5):361‐369. 28457485 10.1016/j.biopsych.2017.03.011 PMC5546993

[51] Carpenter JS , Zmicerevska N , Crouse JJ , et al. Effects of adjunctive brexpiprazole on sleep‐wake and circadian parameters in youth with depressive disorders: study protocol for a clinical trial. BMJ Open. 2022;12(9):e056298. 10.1136/bmjopen-2021-056298 PMC9454051 36691133

[52] Esaki Y , Obayashi K , Saeki K , Fujita K , Iwata N , Kitajima T . Association between circadian activity rhythms and mood episode relapse in bipolar disorder: a 12‐month prospective cohort study. Transl Psychiatry. 2021;11(1):525. 34645802 10.1038/s41398-021-01652-9 PMC8514471

[53] Gershon A , Ram N , Johnson SL , Harvey AG , Zeitzer JM . Daily actigraphy profiles distinguish depressive and interepisode states in bipolar disorder. Clin Psychol Sci. 2015;4(4):641‐650. 27642544 10.1177/2167702615604613 PMC5022043

[54] Stahl EA , Breen G , Forstner AJ , et al. Genome‐wide association study identifies 30 loci associated with bipolar disorder. Nat Genet. 2019;51(5):793‐803. 31043756 10.1038/s41588-019-0397-8 PMC6956732

[55] Coleman JRI , Gaspar HA , Bryois J , et al. The genetics of the mood disorder spectrum: genome‐wide association analyses of more than 185,000 cases and 439,000 controls. Biol Psychiatry. 2020;88(2):169‐184. 31926635 10.1016/j.biopsych.2019.10.015 PMC8136147

[56] Mullins N , Forstner AJ , O'Connell KS , et al. Genome‐wide association study of more than 40,000 bipolar disorder cases provides new insights into the underlying biology. Nat Genet. 2021;53(6):817‐829. 34002096 10.1038/s41588-021-00857-4 PMC8192451

[57] Lewis KJS , Richards A , Karlsson R , et al. Comparison of genetic liability for sleep traits among individuals with bipolar disorder I or II and control participants. JAMA Psychiat. 2020;77(3):303‐310. 10.1001/jamapsychiatry.2019.4079 PMC6902167 31751445

[58] Ferguson A , Lyall LM , Ward J , et al. Genome‐wide association study of circadian rhythmicity in 71,500 UK biobank participants and polygenic association with mood instability. EBioMedicine. 2018;35:279‐287. 30120083 10.1016/j.ebiom.2018.08.004 PMC6154782

[59] Inder ML , Crowe MT , Porter R . Effect of transmeridian travel and jetlag on mood disorders: evidence and implications. Aust N Z J Psychiatry. 2016;50(3):220‐227. 26268923 10.1177/0004867415598844

[60] Wehr TA , Goodwin FK , Wirz‐Justice A , Breitmaier J , Craig C . 48‐hour sleep‐wake cycles in manic‐depressive illness: naturalistic observations and sleep deprivation experiments. Arch Gen Psychiatry. 1982;39(5):559‐565. 6124223 10.1001/archpsyc.1982.04290050037008

[61] Chellappa SL , Morris CJ , Scheer FAJL . Circadian misalignment increases mood vulnerability in simulated shift work. Sci Rep. 2020;10(1):18614. 33122670 10.1038/s41598-020-75245-9 PMC7596056

[62] Short MA , Louca M . Sleep deprivation leads to mood deficits in healthy adolescents. Sleep Med. 2015;16(8):987‐993. 26141007 10.1016/j.sleep.2015.03.007

[63] Scott JPR , McNaughton LR , Polman RCJ . Effects of sleep deprivation and exercise on cognitive, motor performance and mood. Physiol Behav. 2006;87(2):396‐408. 16403541 10.1016/j.physbeh.2005.11.009

[64] Babson KA , Trainor CD , Feldner MT , Blumenthal H . A test of the effects of acute sleep deprivation on general and specific self‐reported anxiety and depressive symptoms: an experimental extension. J Behav Ther Exp Psychiatry. 2010;41(3):297‐303. 20231014 10.1016/j.jbtep.2010.02.008 PMC2862829

[65] Geoffroy PA , Bellivier F , Scott J , Etain B . Seasonality and bipolar disorder: a systematic review, from admission rates to seasonality of symptoms. J Affect Disord. 2014;168:210‐223. 25063960 10.1016/j.jad.2014.07.002

[66] Medici CR , Vestergaard CH , Hadzi‐Pavlovic D , Munk‐Jørgensen P , Parker G . Seasonal variations in hospital admissions for mania: examining for associations with weather variables over time. J Affect Disord. 2016;205:81‐86. 27423064 10.1016/j.jad.2016.06.053

[67] Esaki Y , Obayashi K , Saeki K , Fujita K , Iwata N , Kitajima T . Preventive effect of morning light exposure on relapse into depressive episode in bipolar disorder. Acta Psychiatr Scand. 2021;143:328‐338. 33587769 10.1111/acps.13287

[68] Proudfoot J , Whitton A , Parker G , Doran J , Manicavasagar V , Delmas K . Triggers of mania and depression in young adults with bipolar disorder. J Affect Disord. 2012;143(1):196‐202. 22884233 10.1016/j.jad.2012.05.052

[69] Baethge C , Hennen J , Khalsa H‐MK , Salvatore P , Tohen M , Baldessarini RJ . Sequencing of substance use and affective morbidity in 166 first‐episode bipolar I disorder patients. Bipolar Disord. 2008;10(6):738‐741. 18837869 10.1111/j.1399-5618.2007.00575.x

[70] Tijssen MJ , Van Os J , Wittchen HU , Lieb R , Beesdo K , Wichers M . Risk factors predicting onset and persistence of subthreshold expression of bipolar psychopathology among youth from the community. Acta Psychiatr Scand. 2010;122(3):255‐266. 20199490 10.1111/j.1600-0447.2010.01539.x

[71] Smedler E , Sparding T , Hattab A , Sellgren CM , Landén M . Reporting trigger factors for (hypo)manic episodes in bipolar disorder: association with personality and prognosis. Acta Psychiatr Scand. 2020;141(6):534‐540. 32306385 10.1111/acps.13174

[72] Kenna HA , Poon AW , de los Angeles CP , Koran LM . Psychiatric complications of treatment with corticosteroids: review with case report. Psychiatry Clin Neurosci. 2011;65(6):549‐560. 22003987 10.1111/j.1440-1819.2011.02260.x

[73] Jones I , Craddock N . Familiality of the puerperal trigger in bipolar disorder: results of a family study. Am J Psychiatry. 2001;158(6):913‐917. 11384899 10.1176/appi.ajp.158.6.913

[74] Lewis KJS , Di Florio A , Forty L , et al. Mania triggered by sleep loss and risk of postpartum psychosis in women with bipolar disorder. J Affect Disord. 2018;225:624‐629. 28889048 10.1016/j.jad.2017.08.054

[75] Viguera AC , Tondo L , Koukopoulos AE , Reginaldi D , Lepri B , Baldessarini RJ . Episodes of mood disorders in 2,252 pregnancies and postpartum periods. Am J Psychiatry. 2011;168(11):1179‐1185. 21799064 10.1176/appi.ajp.2011.11010148

[76] Yolken R , Adamos M , Katsafanas E , et al. Individuals hospitalized with acute mania have increased exposure to antimicrobial medications. Bipolar Disord. 2016;18(5):404‐409. 27425597 10.1111/bdi.12416 PMC5508736

[77] Murray KO , Resnick M , Miller V . Depression after infection with West Nile virus. Emerg Infect Dis. 2007;13(3):479‐481. 17552106 10.3201/eid1303.060602 PMC2725905

[78] Yates WR , Gleason O . Hepatitis C and depression. Depress Anxiety. 1998;7(4):188‐193. 9706456 10.1002/(sici)1520-6394(1998)7:4<188::aid-da7>3.0.co;2-6

[79] Lipkin WI , Hornig M . Psychotropic viruses. Curr Opin Microbiol. 2004;7(4):420‐425. 15358262 10.1016/j.mib.2004.06.012

[80] Okusaga O , Yolken RH , Langenberg P , et al. Association of seropositivity for influenza and coronaviruses with history of mood disorders and suicide attempts. J Affect Disord. 2011;130(1–2):220‐225. 21030090 10.1016/j.jad.2010.09.029 PMC3043161

[81] Kennedy SH , Kutcher SP , Ralevski E , Brown GM . Nocturnal melatonin and 24‐hour 6‐sulphatoxymelatonin levels in various phases of bipolar affective disorder. Psychiatry Res. 1996;63(2–3):219‐222. 8878318 10.1016/0165-1781(96)02910-1

[82] Kennedy SH , Tighe S , McVey G , Brown GM . Melatonin and cortisol "switches" during mania, depression, and euthymia in a drug‐free bipolar patient. J Nerv Ment Dis. 1989;177(5):300‐303. 2708973 10.1097/00005053-198905000-00009

[83] Shen GH , Alloy LB , Abramson LY , Sylvia LG . Social rhythm regularity and the onset of affective episodes in bipolar spectrum individuals. Bipolar Disord. 2008;10(4):520‐529. 18452448 10.1111/j.1399-5618.2008.00583.x PMC4090015

[84] Malkoff‐Schwartz S , Frank E , Anderson B , et al. Stressful life events and social rhythm disruption in the onset of manic and depressive bipolar episodes: a preliminary investigation. Arch Gen Psychiatry. 1998;55(8):702‐707. 9707380 10.1001/archpsyc.55.8.702

[85] Malkoff‐Schwartz S , Frank E , Anderson BP , et al. Social rhythm disruption and stressful life events in the onset of bipolar and unipolar episodes. Psychol Med. 2000;30(5):1005‐1016. 12027038 10.1017/s0033291799002706

[86] McKnight RF , de La Motte de Broöns de Vauvert SJGN , Chesney E , Amit BH , Geddes J , Cipriani A . Lithium for acute mania. Cochrane Database Syst Rev. 2019;6(6):CD004048. 31152444 10.1002/14651858.CD004048.pub4 PMC6544558

[87] Kishi T , Ikuta T , Matsuda Y , et al. Pharmacological treatment for bipolar mania: a systematic review and network meta‐analysis of double‐blind randomized controlled trials. Mol Psychiatry. 2021;26:4146‐4157. 34642461 10.1038/s41380-021-01334-4 PMC9054678

[88] Yatham LN , Kennedy SH , Parikh SV , et al. Canadian network for mood and anxiety treatments (CANMAT) and International Society for Bipolar Disorders (ISBD) 2018 guidelines for the management of patients with bipolar disorder. Bipolar Disord. 2018;20(2):97‐170. 29536616 10.1111/bdi.12609 PMC5947163

[89] Severus E , Taylor MJ , Sauer C , et al. Lithium for prevention of mood episodes in bipolar disorders: systematic review and meta‐analysis. Int J Bipolar Disord. 2014;2(1):15. 25530932 10.1186/s40345-014-0015-8 PMC4272359

[90] Geddes JR , Burgess S , Hawton K , Jamison K , Goodwin GM . Long‐term lithium therapy for bipolar disorder: systematic review and meta‐analysis of randomized controlled trials. Am J Psychiatry. 2004;161(2):217‐222. 14754766 10.1176/appi.ajp.161.2.217

[91] Malhi GS , Outhred T . Therapeutic mechanisms of lithium in bipolar disorder: recent advances and current understanding. CNS Drugs. 2016;30(10):931‐949. 27638546 10.1007/s40263-016-0380-1

[92] Federoff M , McCarthy MJ , Anand A , et al. Correction of depression‐associated circadian rhythm abnormalities is associated with lithium response in bipolar disorder. Bipolar Disord. 2022;24(5):521‐529. 34825444 10.1111/bdi.13162

[93] Barbini B , Benedetti F , Colombo C , et al. Dark therapy for mania: a pilot study. Bipolar Disord. 2005;7(1):98‐101. 15654938 10.1111/j.1399-5618.2004.00166.x

[94] Henriksen TE , Skrede S , Fasmer OB , et al. Blue‐blocking glasses as additive treatment for mania: a randomized placebo‐controlled trial. Bipolar Disord. 2016;18(3):221‐232. 27226262 10.1111/bdi.12390 PMC5089565

[95] Phelps J . Dark therapy for bipolar disorder using amber lenses for blue light blockade. Med Hypotheses. 2008;70(2):224‐229. 17637502 10.1016/j.mehy.2007.05.026

[96] Esaki Y , Takeuchi I , Tsuboi S , Fujita K , Iwata N , Kitajima T . A double‐blind, randomized, placebo‐controlled trial of adjunctive blue‐blocking glasses for the treatment of sleep and circadian rhythm in patients with bipolar disorder. Bipolar Disord. 2020;22:739‐748. 32276301 10.1111/bdi.12912

[97] Robillard R , Naismith SL , Rogers NL , et al. Delayed sleep phase in young people with unipolar or bipolar affective disorders. J Affect Disord. 2013;145(2):260‐263. 22877966 10.1016/j.jad.2012.06.006

[98] Byrne EM , Heath AC , Madden PAF , et al. Testing the role of circadian genes in conferring risk for psychiatric disorders. Am J Med Genet B Neuropsychiatr Genet. 2014;165(3):254‐260. 10.1002/ajmg.b.32230 PMC4397914 24687905

[99] Pagani L , St Clair PA , Teshiba TM , et al. Genetic contributions to circadian activity rhythm and sleep pattern phenotypes in pedigrees segregating for severe bipolar disorder. Proc Natl Acad Sci U S A. 2016;113(6):E754‐E761. 26712028 10.1073/pnas.1513525113 PMC4760829

[100] Geoffroy PA , Lajnef M , Bellivier F , et al. Genetic association study of circadian genes with seasonal pattern in bipolar disorders. Sci Rep. 2015;5:10232. 25989161 10.1038/srep10232 PMC4437291

